# Dysphagia and pill swallowing in HIV/AIDS in South Africa: Results of a scoping review

**DOI:** 10.4102/sajcd.v70i1.955

**Published:** 2023-03-31

**Authors:** Alexa Cohen, Jaishika Seedat, Cynthia Sawasawa

**Affiliations:** 1 Department of Speech Pathology and Audiology, Faculty of Humanities, University of the Witwatersrand, Johannesburg, South Africa

**Keywords:** HIV, AIDS, HAART, adult dysphagia, pill swallowing, South Africa, primary healthcare, SLP

## Abstract

**Background:**

South Africa has the highest prevalence of human immunodeficiency virus (HIV) worldwide. Highly active antiretroviral therapy (HAART) is expected to improve the quality of life for these individuals but requires long-term medication intake. Poor pill adherence and related dysphagia are undocumented for individuals on HAART regimens living in South Africa.

**Aim:**

To conduct a scoping review describing the presentation of pill swallowing difficulties and dysphagia experiences of individuals with HIV and acquired immunodeficiency syndrome (AIDS) in South Africa.

**Method:**

This review describes the presentation of pill swallowing difficulties and dysphagia experiences of individuals with HIV and AIDS in South Africa using a modified version of the Arksey and O’Malley framework. Five search engines targeting published journal articles were reviewed. Two hundred and twenty-seven articles were retrieved; however, following the exclusion criteria based on PICO, only three articles were included. Qualitative analysis was completed.

**Results:**

The reviewed articles identified swallowing difficulties that adults with HIV and AIDS experienced and confirmed non-adherence to medical regimens. Barriers and facilitators of pill swallowing with dysphagia due to the side-effects of the pill itself were documented with physical properties of the pill not influencing adherence.

**Conclusion:**

The speech-language pathologists (SLPs) role with individuals with HIV/AIDS to facilitate improved pill adherence was lacking with limited research on the management of swallowing difficulties in this population. The review identified dysphagia and pill adherence management by the SLP in South Africa as caveats for further research.

**Contribution:**

Speech-language pathologists must monitor swallowing during mealtimes as well as pill swallowing in individuals with HIV/AIDS due to the compromise of their oral health and oral structures. Speech-language pathologists therefore have to advocate for their role in the team managing this population of patients. Their involvement may reduce the risk of nutritional compromise as well as patient non-compliance with medication stemming from pain and inability to swallow solid oral dosage forms of medication.

## Background

Although South Africa (SA) is classified as an upper-middle income country, it still faces unique public health challenges associated with a quadruple burden of disease (Pillay-Van Wyk et al., 2017). Despite the remarkable improvement in the initiatives targeting human immunodeficiency virus (HIV) prevention and improved accessibility to highly active antiretroviral therapy (HAART) for treatment, it remains a health challenge (Biney et al., 2020). An estimated 7.5 million (18%) people are living with HIV and/or acquired immunodeficiency syndrome (AIDS) in SA, making it the country with the highest prevalence in the world (Statistics South Africa, 2020). Consequently, SA has the largest HAART treatment programme which has reduced fatalities and incidence of opportunistic infections among people living with HIV (Meintjes et al., 2017), increasing their life expectancy and improving their quality of life (QoL).

Highly active antiretroviral therapy is a long treatment regime that consists of a combination of three or more antiretroviral drugs (Ford et al., 2018) to inhibit viral replication, reduce the transmission of HIV, improve immune function and prevent drug resistance (Thompson et al., 2012). Moreover, it consists of either a combination pill taken daily, or several pills taken twice daily (Myhre & Sifris, 2020). It is recommended that HAART commence within 7 days of confirmed HIV diagnosis (Eggleton & Nagalli, 2022). Due to the inability of HAART to completely destroy the virus, treatment is typically lifelong (Henderson et al., 2019); hence, adherence is critical. Regrettably, poor adherence to HAART prevails (Myhre & Sifris, 2020), with pill burden and dysphagia (Azia et al., 2016) being major contributors.

Dysphagia is characterised by the inability or difficulty swallowing (Badger & Verma, 2018). Although swallowing happens in phases, being a continuous process, any difficulty or impairment in one of the phases, that is, oral, pharyngeal or oesophageal can influence the others, inadvertently impacting the entire swallowing process (Soutinho et al., 2021). There are various causes of dysphagia (Badger & Verma, 2018) leading to negative health consequences such as malnutrition, dehydration and aspiration pneumonia (Dobak & Kelly, 2021). Due to opportunistic infections such as oesophageal candidiasis, the herpes simplex virus, and aphthous ulcerations, dysphagia is common in this population. The presentation of dysphagia is thus characterised by reduced awareness and sensitivity in the pharynx causing adverse consequences such as choking, coughing or aspiration. Dysphagia may also be induced by the physical properties such as size and texture of pills (Radhakrishnan et al., 2021) or the side-effects of the drug composition of pills (Fields et al., 2015). These may result in the dysfunction of the swallowing mechanism as a whole (all phases) due to the effect of the medication, but there may be specific effects on the smooth or striated muscle function of the oesophagus (Fields et al., 2015).

The design and purpose of pills is not particularly focused on making the pills easy to swallow, but rather to ensure beneficial medical outcomes, hence physical properties of pills remain a common source of frustration among individuals. Larger pills require multiple swallows to clear the oesophagus and are prone to causing pill-induced dysphagia (Radhakrishnan et al., 2021). The effects of pill swallowing difficulties can range from mild discomfort to life threatening complications such as aspiration.

Speech-language pathologists (SLP) assess and manage dysphagia within a team of other individuals. Over and above limited knowledge about the role of the SLP in the treatment of pill-induced dysphagia for people living with HIV/AIDS (Alborough, 2012), there is sparse information available about pill-swallowing difficulties, dysphagia and the subsequent effect on pill adherence in this population.

## Method

This review described the presentation of pill-swallowing and dysphagia experiences of individuals with HIV/AIDS in SA, specifically documenting how pill adherence is affected by pill swallowing difficulties and dysphagia among individuals with HIV/AIDS, the presentation of their experiences, the impact their swallowing difficulties and dysphagia have on their adherence to medication, and the possible involvement of the SLP in the treatment and management of pill swallowing and dysphagia. The participants in the articles were individuals who were diagnosed with HIV and/or AIDS living in SA and presenting with difficulty swallowing pills.

Five databases were used (Table 1) with search terms specific to each database. Journal articles, theses and dissertations published from 2000 until 2020 were reviewed. Keywords, titles and abstracts of all selected articles were analysed. No additional articles emerged from reviewing the reference list of each selected article. Descriptive statistics identified characteristics that could influence the outcomes affecting the research in a meaningful way. Thematic content analysis examined the ways in which events, realities and experiences manifested.

**TABLE 1:** Search terms and databases included in the scoping review.

Database	Search term
CiNAHL	Adults and HIV or AIDS and ARV and Pill adherence and South Africa
EBSCO Africa Wide	Pill non-adherence and adults and South Africa and HIV or AIDS and swallowing difficulties
PubMed	South Africa and HIV or AIDS and pill adherence and dysphagia and adults
SABINET African Journals	South Africa and HIV or AIDS and pill non-adherence and adults
Scopus	Adults and HIV or AIDS and dysphagia and South Africa

HIV, human immunodeficiency virus; AIDS, acquired immunodeficiency syndrome; ARV, antiretroviral.

### Ethical considerations

An ethics waiver was obtained from the University of the Witwatersrand Human Research and Ethics Committee (Non-Medical) (Waiver Number STA_2021_3W).

## Results

**FIGURE 1:**
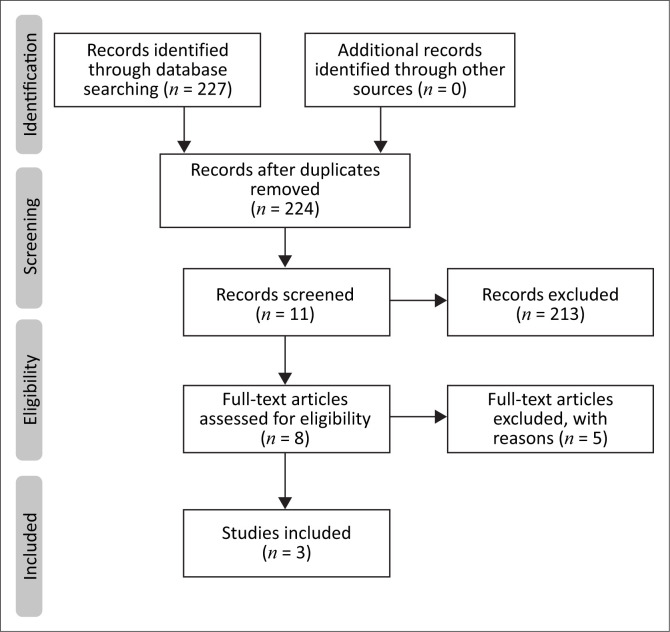
Preferred reporting items for systematic reviews and meta-analysis flow diagram for scoping reviews (PRISMA-ScR).

Figure 1 provides the completion of the Preferred Reporting Items for Systematic Reviews and Meta-Analysis flow diagram modified for the scoping review process (PRISMA-ScR) from the Joanna Briggs Institute (Peter et al., 2019). It ensured applicable and appropriate recordings of data.

As seen in Figure 1, 227 records were identified from the five databases. Duplicates were removed (*n* = 3) leaving 224 articles. Titles and abstracts were screened for eligibility against the inclusion and exclusion criteria. Two hundred and thirteen articles were excluded as they did not meet the inclusion criteria, leaving the researcher with 11 articles. Eight articles were not relevant leaving three articles for inclusion in the scoping review.

Table 2 highlights the major themes that emerged from analysis of the articles (Bladon & Ross, 2007; Naidoo, 2009; Peltzer & Phaswana-Mafuya, 2008).

**TABLE 2:** Emergent themes in relation to the objectives of this study.

Objective	Theme
1. To document the reported barriers and facilitators of pill swallowing experienced by the HIV and AIDS population.	Barriers Side-effects of HAART Co-existing medical symptoms Personal factors (contextual limitation and psychosocial factors) Facilitators Personal variables (understanding the diagnosis and access) Management strategies
2. To explain how dysphagia or swallowing difficulties directly impacts pill adherence among the individuals with HIV and AIDS.	Physical dysfunction Oral intake
3. To determine the involvement of the SLP in the management of individuals with HIV and AIDS.	Late involvement due to limited knowledge of the role of the SLP

HIV, human immunodeficiency virus; AIDS, acquired immunodeficiency syndrome; HAART, highly active antiretroviral therapy; SLP, speech-language pathologist.

### Objective 1 (part 1): Barriers to pill swallowing

#### Side-effects of highly active antiretroviral therapy

From the three articles, 55% of participants identified side-effects of HAART such as nausea, dizziness and insomnia as barriers to pill swallowing. Complex regimens with multiple pills each with their own ingredients also affected pill swallowing. Seventy nine point two percent of participants reported side-effects of HAART medication, such as dry mouth, nausea and sores developing in the mouth or throat. Fifty nine point three percent of the sample in the study by Peltzer and Phaswana-Mafuya (2008) reduced their food intake due to the medication side-effects.

#### Co-existing medical symptoms

All three articles referred to dysphagia resulting from comorbidities that contributed to the inability or difficulty in swallowing pills. Naidoo (2009) identified the herpes simplex virus, hepatitis, diabetes, lipodystrophy or a low CD4 count as contributory factors to difficulty swallowing pills and ultimately non-adherence. In comparison, Bladon and Ross (2007) identified candidiasis of the oral cavity and upper digestive tract, herpes simplex virus infections such as small, painful, fluid-filled wounds in and around the mouth, gastro-oesophageal reflux disease or oesophageal dysmotility as impeding medication adherence.

Participants reported odynophagia (i.e. pain while swallowing), across the stages of the disease which impacted adherence (Peltzer & Phaswana-Mafuya, 2008). This could potentially lead to reduced oral intake of essential liquids and foods. They established that malnutrition had a negative effect on the immune system resulting in a decline of CD4 T-cells, suppression of delayed hypersensitivity and abnormal B-cell responses. As a result, the individual with HIV/AIDS was susceptible to a higher risk of opportunistic infections causing symptoms and side-effects that were detrimental to pill swallowing or swallowing in general.

#### Personal factors

**Contextual limitations:** Naidoo (2009) found two main reasons for poor medication adherence. (1) working far from their residence and regularly forgetting to take medication and (2) not being included in the decision-making process and so unwilling to take the medication. In addition, Peltzer and Phaswana-Mafuya (2008) highlighted age, low education level, insufficient food intake due to contextual factors and reliance on a government-funded grant as indicators for non-adherence.

**Psychosocial factors:** All three articles linked psychological and social factors as barriers to pill swallowing. Socially, the stigma attached to HIV/AIDS impacted adherence to HAART (Naidoo, 2009). Non-adherence was associated with younger age and more specifically among young females (Naidoo, 2009). Body pain and odynophagia contributed to anxiety impacting swallowing (Bladon & Ross, 2007). Similarly, Peltzer and Phaswana-Mafuya (2008) reported that participants experienced high levels of psychological stress such as fear, concern, depression and anxiety when dealing with their HIV/AIDS status, which impacted their pill compliance.

### Objective 1 (part 2): Facilitators to pill swallowing

#### Personal factors

**Understanding the diagnosis:** Eighty three point six percent of participants stated that when they were provided sufficient information on why medication was necessary and the procedure for correct medication intake, it increased their confidence making them more comfortable and compliant with their regimes (Naidoo, 2009). Furthermore, information provided by their healthcare professionals about HIV/AIDS facilitated understanding, leading them to accept their diagnosis and comply with the medical recommendations (Naidoo, 2009).

**Access:** Naidoo (2009) reported that reliable access to healthcare professionals and information improved dosage schemes with participants experiencing less adverse and undesirable medication effects, ultimately improving pill compliance. Similarly, Peltzer and Phaswana-Mafuya (2008) found that participants’ access to conducive living conditions and environments, as well as nutrition, that is, food security, assisted in the management of symptoms related to HIV/AIDS and this resulted in a positive response to the prescribed medical treatment.

#### Management strategies

All articles provided strategies to circumvent non-adherence to medication. Naidoo (2009) suggested a reminder system such as a short message services on one’s cell phone or setting an alarm on wrist watches to prompt medication intake. Bladon and Ross (2007) documented that 58.9% of their participants used ‘self-help’ techniques to reduce their swallowing difficulties such as drinking more water and avoiding solid foods and foods that induce heartburn. Compensatory techniques such as cyclic ingestion, bolus placement and using head extension during a swallow for gravity to propel the bolus into the pharynx (Bladon & Ross, 2007) were also used. Some participants identified antiacids effective in relieving side-effects of the medication.

### Objective 2: How dysphagia or swallowing difficulties affect pill adherence

#### Physical dysfunction

Naidoo (2009) highlighted that participants’ who experienced symptoms of dysphagia were physically unable to swallow the pills. Similarly, Bladon and Ross (2007) identified dysphagia and odynophagia as common complaints at all stages of the disease. This resulted in discomfort during the swallow, increasing anxiety. Reports pertaining to salivary gland function were noted (Bladon & Ross, 2007) as saliva is integral to swallowing, aiding in lubrication, bolus formation and bolus propulsion. Participants with hyposalivation reported increased pain and difficulty while swallowing, consequently affecting their ability to comply with their medical regimens. Consequently, participants’ reduced oral intake to avoid pain and discomfort adversely impacted nutritional intake and ultimately adherence (Bladon & Ross, 2007). Thus, swallowing difficulties meant poor pill adherence and a consequent decrease in CD4 count resulting in more severe swallowing difficulties, hence a vicious cycle.

#### Oral intake

Highly active antiretroviral therapy treatment regimens are complex and require individuals to take a large quantity of pills on a full stomach. Participants whose medical regimes required them to complete a full meal while also experiencing symptoms of dysphagia were not able to comply with pill adherence (Naidoo, 2009).

### Objective 3: The involvement of the speech-language pathologists in the management of HIV and AIDS

#### Late involvement due to limited knowledge of the role of the speech-language pathologists

Despite the link between pill swallowing and swallowing difficulty, the SLP featured very little, if at all in the reviewed articles. While featured in the article by Bladon and Ross (2007) this was likely because Bladon is a SLP. They noted that when SLPs are involved in management, it is generally at later stages of disease presentation when the swallowing difficulty is severe and more explicit. Importantly, management by the SLP positively impacted nutritional intake and QoL. It is likely that healthcare professionals may be unaware of the full extent of the role of the SLP given that it featured in only one article.

## Discussion

With only three articles in the final review, it is clear that there is limited research on pill adherence in relation to dysphagia among individuals with HIV/AIDS in SA. The role and involvement of the SLP with this population is scarce and was found in one article only. While the exact number of individuals with HIV/AIDS who experience dysphagia and pill swallowing difficulty is unknown, all the articles noted a high percentage, confirming the need for more research in this area. When SLPs are only involved in the later stages of HIV/AIDS undesirable consequences are more likely (Bladon & Ross, 2007) having a knock-on effect on individuals not adhering to their medical regimens and reducing their QoL (Malouh et al., 2020).

Notwithstanding the limited literature on the role of SLP in the assessment and treatment of dysphagia in this population (Warner, 2015), existing data confirms the need for SLP involvement to manage additional pathologies that are known to occur as a direct result of HIV/AIDS. Earlier intervention of dysphagia is believed to improve the therapeutic and medical outcomes and overall QoL of individuals (Nel & Ellis, 2012). Malouh et al. (2020) highlighted that the role of the SLP with pill swallowing was not acknowledged to be within the scope of dysphagia management for the SLP while the HIV/AIDS pandemic was rife in SA during the 1980s – 1990s. However, it is likely that other professionals may not have recognised the relationship between the role of the SLP and pill swallowing (Malouh et al., 2020) compounding late referral and involvement of SLPs (Jackson et al., 2008). Without the involvement of the SLP, individuals remain unaware about dysphagia and the reasons contributing to their poor adherence (Seedat & Zayannakis, 2020). Thus, doctors continue to prescribe medication that the individual cannot adhere to. This vicious cycle has the ability to unnecessarily alter an individual’s QoL.

The World Health Organization (WHO) defines QoL as an individual’s overall welfare and safety in relation to their physical health, psychological well-being, social affiliations and the environment in which they live in (WHO, 2014). When ensuring QoL of an individual, one turns to the International Classification of Functioning, as it defines the prominent relationship between dysphagia and contextual factors of the individual. It allows the SLP and other healthcare professionals to specify how dysphagia has the ability to cause harmful and undesirable effects on the individual’s QoL. These include a decline in nutritional intake, weight loss or increased risk for secondary infections (Warner, 2015). These effects can lead to an individual resorting to social and complete isolation (Bladon & Ross, 2007). Quality of life is affected when the individual’s activities and participation in daily living are decreased compared to their premorbid way of life, purely as a result of their health condition (Warner, 2015).

In addition to the presence of dysphagia impacting QoL, it is important for all healthcare professionals, including the SLP to be cognisant of how pills may contribute to, and exacerbate, an already existing dysphagia, or else present as a pill induced dysphagia. The physical properties (size, shape, texture, coating), chemical make-up, and the side-effects of the pills may further adversely impact existing dysphagia (Radhakrishnan et al., 2021). For individuals with HIV/AIDS, this is especially relevant given the varying pills needed. Highly active antiretroviral therapy reduces viral load, increases immunity and resistance to the virus (Myhre & Sifris, 2020), thus when not taken, the effects may be detrimental. It is therefore important that the SLP advocates their role within all populations where pill swallowing and medication intake form a major part of their progress and recovery. This review confirms the need for more specific research on pill adherence in the HIV/AIDS population, their lived experience of pill swallowing, and the influence that SLP intervention has on pill swallowing and adherence.

## Conclusion

Dysphagia is an emerging and promising field, and while research on the HIV/AIDS population is increasing, more is needed. While this study was specific to pill swallowing difficulties in this population, pill swallowing difficulty is not selective. Research implications for other populations dependent on medication intake warrants investigation.

The limited research on the role of SLPs in the management of pill swallowing difficulties of individuals with HIV/AIDS needs to change. It is possible that if individuals with HIV/AIDS are not adhering to their medication due to reasons pertaining to dysphagia – whether it be pill-induced or typical dysphagia; there are implications for further referral, consultation and collaboration between SLPs in a multidisciplinary team which may ensure optimal and well-rounded care for individuals with HIV/AIDS.

### Limitations

During this scoping review a limitation was noted. Quality of life is not typically covered in literature when looking at the management of a disease, such as HIV and AIDS, specifically in the management of pill adherence. The impact of swallowing pills does cover the effects on QoL and hence it should be something that future researchers conducting reviews are aware of. Additionally, having a broader scoping review that looked at Africa as a whole and not specifically SA would have allowed the researchers to work with a larger range of literature pertaining to the subject. This is therefore also recommended for future reviews in this area.
